# Therapeutic Strategies against Ebola Virus Infection

**DOI:** 10.3390/v14030579

**Published:** 2022-03-11

**Authors:** Ching-Hsuan Liu, Yee-Tung Hu, Shu Hui Wong, Liang-Tzung Lin

**Affiliations:** 1 Department of Microbiology and Immunology, School of Medicine, College of Medicine, Taipei Medical University, Taipei 110, Taiwan; d119107007@tmu.edu.tw; 2 Graduate Institute of Medical Sciences, College of Medicine, Taipei Medical University, Taipei 110, Taiwan; d119110004@tmu.edu.tw; 3 International Ph.D. Program in Medicine, College of Medicine, Taipei Medical University, Taipei 110, Taiwan; d142109016@tmu.edu.tw

**Keywords:** Ebola virus, Ebola virus disease, antiviral, therapeutic, drug development

## Abstract

Since the 2014–2016 epidemic, Ebola virus (EBOV) has spread to several countries and has become a major threat to global health. EBOV is a risk group 4 pathogen, which imposes significant obstacles for the development of countermeasures against the virus. Efforts have been made to develop anti-EBOV immunization and therapeutics, with three vaccines and two antibody-based therapeutics approved in recent years. Nonetheless, the high fatality of Ebola virus disease highlights the need to continuously develop antiviral strategies for the future management of EBOV outbreaks in conjunction with vaccination programs. This review aims to highlight potential EBOV therapeutics and their target(s) of inhibition, serving as a summary of the literature to inform readers of the novel candidates available in the continued search for EBOV antivirals.

## 1. Introduction

Since the discovery of Ebola virus and Sudan virus in 1976 [1], ebolaviruses have become a significant public health threat. Ebolaviruses (members of the genus *Ebolavirus*) belong to the *Filoviridae* family and comprise six species: Ebola virus (EBOV), Sudan virus (SUDV), Reston virus (RESTV), Bundibugyo virus (BDBV), Taï Forest virus (TAFV), and Bombali virus (BOMV) [2]. While EBOV, SUDV, and BDBV cause fatal human infections, with EBOV being the most virulent and the leading cause of most outbreaks, RESTV has only caused an asymptomatic human infection, TAFV has only caused a single case of non-lethal human disease, and no human cases of BOMV have been reported [1,2]. EBOV caused several localized outbreaks in Middle Africa (the Democratic Republic of the Congo [DRC], Gabon, and the Republic of the Congo), before the unprecedented 2014–2016 West African outbreak began in Guinea and ultimately spread to over 15 countries, leading to 28,652 cases and 11,325 deaths [1,3]. Several factors facilitated the expansion of the 2014–2016 EBOV outbreak, such as international dispersal, urbanization (the population size of origins and destinations and shorter travel times), the absence of preparedness of both the exposed population and the international community [4], and adaptive viral mutations [5]. Since the 2014–2016 EBOV epidemic, additional outbreaks of EBOV have occurred, including the 2018–2020 outbreak in the DRC (3481 cases and 2299 deaths), the 2021 outbreak in Guinea, and the ongoing outbreak in DRC since 2021 which is linked to the 2018–2020 outbreak [6], highlighting the need for continued vigilance.

EBOV is highly contagious and can be transmitted through direct contact with blood or body fluids from infected individuals, fomites, and infected wild animals [7]. The virus RNA has also been detected in breast milk, vaginal secretions, and the semen of convalescent patients, providing evidence of sexual transmission [8]. EBOV infection in humans causes Ebola virus disease (EVD). The most common manifestations of EVD during the 2014–2016 outbreak included fatigue, anorexia, abdominal pain, diarrhea, vomiting, fever, and myalgia, and the overall case fatality rate was approximately 40% [3]. To date, there are three approved EBOV vaccines, including one vesicular stomatitis virus (VSV)-based vector and two adenovirus-based vectors. The VSV-based vector, Ervebo^®^ (rVSV-ZEBOV), is a replication-competent recombinant vaccine engineered to express EBOV glycoprotein (GP). Ervebo^®^ received regulatory approval for use in Europe [9] and the United States [10] in 2019, as well as in four African countries (DRC, Burundi, Ghana, and Zambia) in 2020 [11]. The adenovirus-based vaccines, Zabdeno^®^/Mvabea^®^ (a two-dose regimen containing Ad26.ZEBOV and MVA-BN-Filo) and Ad5-EBOV, have been approved in Europe and China, respectively [12]. Meanwhile, the antibody-based therapeutics Inmazeb™ (i.e., REGN-EB3) [13] and Ebanga™ (i.e., mAb114) [14] received approval as treatments for EBOV in 2020. Nonetheless, the high fatality rate of EVD indicates that the continuous development of antivirals is necessary to improve its current management and increase preparedness and vigilance for future emergencies.

EBOV is an enveloped filamentous virus containing a non-segmented negative-sense single-stranded RNA genome. The approximately 19 kb genome of the virus encodes nine viral proteins (VPs)—nucleoprotein (NP), polymerase cofactor (VP35), matrix protein (VP40), glycoprotein (GP), secreted glycoprotein (sGP), secondary secreted glycoprotein (ssGP), transcriptional activator (VP30), RNA complex-associated protein (VP24), and large protein (L; polymerase) [1]. The EBOV GP has two subunits: GP1 and GP2. While GP1 is chiefly involved in viral attachment to the host cell receptors, GP2 is mainly responsible for membrane fusion. Following attachment to cell-surface molecules, such as C-type lectins; T cell immunoglobulin mucin (TIM) proteins; and the TYRO3, AXL, and MERTK (TAM) family receptor tyrosine kinases, the virion enters the cell by endocytosis (mainly macropinocytosis) and is trafficked to endolysosome, where the glycan cap on GP is cleaved by the host cysteine proteases cathepsins B and L [1]. The cleavage of the glycan cap exposes the GP receptor binding site, which then binds to Niemann-Pick C1 (NPC-1) in the host cell, a key receptor of EBOV entry. GP1 interacts with NPC-1 to mediate membrane fusion along with GP2 subunits [15]. The viral membrane subsequently fuses with the endosomal membrane to release the viral ribonucleoprotein (RNP) complex into the cytoplasm [16]. Following release, viral genome transcription (mRNA synthesis) is activated by the transcription factor VP30 [17], and viral proteins are translated to form the viral replication machinery in the cytoplasm [18]. Encapsidated antigenomes are synthesized to serve as templates for progeny genome synthesis [1], and VP24 interacts with NP to assist in nucleocapsid formation [19]. Eventually, mature RNPs are transported to the cell membrane for matrix embedding and envelopment, and VP40 induces budding of the viral particles [1]. In addition, VP35 and VP24 also play vital roles in antagonizing the host’s innate immunity. VP35 is capable of (1) binding viral dsRNA to prevent retinoic acid inducible gene-I (RIG-I) recognition and (2) inhibiting the phosphorylation of interferon regulatory factors (IRF)-3 and -7 by the kinases Tank binding kinase-1 (TBK-1) and I-kappa-B kinase epsilon (IKKε) [20], whereas VP24 binds to karyopherin-α (KPN-α) to inhibit nuclear transportation of the phosphorylated signal transducer and activator of transcription (STAT) 1 [21]. Given the significance and the multifunctionality of EBOV proteins in the viral life cycle [1], targeting any of these proteins could constitute a plausible antiviral strategy against the infectious agent. 

## 2. Antiviral Strategies against EBOV

In this review, we will describe EBOV inhibitors targeting various viral proteins and host factors. The stage of development and 50% inhibitory concentration (IC_50_) of each inhibitor are shown in Table 1. In addition, Table 2 summarizes the conditions used and the results observed in animal studies. 

### 2.1. EBOV VP35 Inhibitors

EBOV VP35 is the cofactor protein in the viral polymerase complex [133] and an antagonist against the host’s innate immunity type I interferon (IFN) response [20,134]. In one study, the inhibition of EBOV VP35 with antisense phosphorodiamidate morpholino oligomers (PMO; a single-stranded DNA analog) or PMO conjugated to cell-penetrating peptide (P-PMO) was able to prevent EBOV infection in vitro as well as in mice before and after infection [22]. A few compounds have also been shown to exhibit anti-VP35 activities. These include myricetin from *Limonium morisianum* extract as an inhibitor of the VP35–dsRNA interaction [23], and the small molecule MCCB4 (a linear hydrophobic molecule containing an ene-thiazolidinedione group) which impedes EBOV replication and transcription by blocking the VP35–NP interaction [24]. A recent study further developed anti-VP35 single-chain variable fragment (scFv) intracellular antibodies that could significantly subvert VP35-induced IFN-β suppression [25]; their antiviral activity may be further investigated. 

### 2.2. EBOV VP40 Inhibitors

EBOV VP40 is not only important for virus particle formation [135], but is also involved in viral replication and transcription [136], making it a promising target for EBOV therapy. Cell-penetrable anti-VP40 scFv antibodies have been shown to inhibit EBOV virus-like particles (VLP) budding from human hepatocytes [26]. Small molecules that disrupt the interaction between VP40 and the host Nedd4 ubiquitin ligase, such as quinoxaline-based inhibitors, have also displayed anti-budding activities [27]. On the other hand, an in silico screening of sugar alcohol compounds suggested that sorbitol, mannitol, and galactitol could bind to the VP40 octamer [28], an RNA-binding structure that is crucial for the EBOV life cycle [137,138]. Another in silico study also predicted seven pyrimidinediones class molecules that could block the VP40 Arg134 RNA-binding site [29]. These molecules could potentially interrupt the VP40–RNA interaction; however, their antiviral effects remain to be validated with biological experiments.

### 2.3. EBOV GP Inhibitors

EBOV GP mediates viral entry via its two subunits and is the most extensively studied antiviral target. The importance of GP as a therapeutic target is reflected by (1) the presence of GP-neutralizing antibodies in the plasma of those who were naturally infected [139,140] or vaccinated [41,47], and (2) the effectiveness of the VSV-vectored EBOV-GP vaccine as a post-exposure prophylaxis in animal models [141] as well as in the Guinea ring vaccination trial [142]. Inhibitors of EBOV GP include neutralizing antibodies, synthetic compounds, and natural compounds. GP-specific antibodies appear to be the most effective post-exposure therapeutics based on current literature. Several neutralizing monoclonal antibodies (mAb) have been characterized, and some of them have provided promising effects in the treatment of non-human primates (NHP) after lethal EBOV infections. These include the single antibody mAb114 [30]; the two-antibody cocktail MIL77E (c13C6 and c2G4; derived from ZMapp^TM^) [33]; and the three-antibody cocktails ZMAb (m1H3, m2G4, and m4G7) [100,114,143], MB-003 (c13C6, h13F6 and c6D8) [35,36,37,38], ZMapp^TM^ (c13C6, c2G4, and c4G7; derived from ZMAb and MB-003) [32], and REGN-EB3 (REGN3470, REGN3471, and REGN3479) [31]. When subsequently studied in clinical trials, ZMapp^TM^ was shown to reduce the case mortality rate from 37% (control arm) to 22% [144,145]. Afterwards, mAb114 and REGN-EB3 were evaluated in the 2018–2019 DRC outbreak (the Pamoja Tulinde Maisha trial), where both antibodies demonstrated superior efficacy compared to the control ZMapp^TM^ in reducing EVD mortality [146]. Based on the trial results, REGN-EB3 (Inmazeb™) and mAb114 (Ebanga™) were both approved as treatments for EBOV infection [13,14]. The effect of other EBOV GP specific mAbs, such as KZ52 [39,118], Q206, Q314, Q411 [40], 2G1, 5E1, and 5E9 [41], are summarized in Table 1 and Table 2. 

Since other ebolaviruses may also lead to fatal human infections, several recent studies have aimed to produce cross-protective antibodies that can neutralize multiple ebolaviruses, i.e., broadly neutralizing antibodies (bNAb). For example, the mAb 6D6 [42] and the bispecific antibodies (Bis-mAbs) [44] have been shown to be protective in mice. FVM04 [46], CA45 [45,119], and the four-antibody cocktail (040 + 66-3-9C + 6662 + 6541) [47] were shown to protect guinea pigs. Furthermore, the antibody cocktails FVM04 + CA45 [119], MBP134^AF^ (ADI-15878 and ADI-23774) [120,121], and rEBOV-520 + rEBOV-548 [49] were all shown to fully protect NHP from lethal EBOV challenge.

In addition to mAbs, cost-effective polyclonal antibodies (pAb) with a higher production yield, such as IgG (EBOV/SUDV pAb) purified from immunized transchromosomal bovines [50] and polyclonal fragments F(ab’)_2_ produced from hyperimmunized horses [51], have also demonstrated post-exposure protection in mice and NHP, respectively. 

Multiple GP-targeted compounds have also been explored. Recombinant human mannose-binding lectin (rhMBL) has been shown to block EBOV binding to the attachment factor DC-SIGN (dendritic cell-specific intercellular adhesion molecule-3-grabbing non-integrin) [147], and rhMBL reconstitution therapy was shown to protect 40% of treated mice from fatal EBOV infection and rechallenge [52]. 3-hydroxyphthalic anhydride (HP)-modified human serum albumin (HSA) (HP-HSA) has also demonstrated its ability to bind to GP and block EBOV attachment [55]. Furthermore, the modified protein is highly thermostable for storage and remains effective after 8 weeks at 45 °C, which is advantageous to its potential real-world application, especially in tropical African regions [55]. Polyphenylene carboxymethylene (PPCM), a polymer derived from mandelic acid condensation that displays broad-spectrum vaginal microbial activities, was shown to inhibit EBOV GP attachment as well [56]; thus, it may be useful for preventing the sexual transmission of EBOV. Several FDA-approved drugs, including clomiphene, toremifene [53], bepridil, and sertraline [54], were found to inhibit EBOV fusion and protect mice from lethal EBOV infection. Computational and biophysical analyses indicated that these drugs could directly bind to GP and destabilize the pre-fusion structure, thereby preventing its fusion with the endosomal membrane [148,149,150]. Some natural compounds, such as sclareol and sclareolide derived from *Salvia sclarea*, were also found to inhibit EBOV viral fusion [57]. Finally, several compounds were predicted to bind to GP and could inhibit EBOV pseudoparticle entry, including the benzodiazepine derivative compound **7** [58], two derivatives of natural isoflavones ZINC32540717 (compound **118**) and ZINC09410451 (compound **118a**) [59], and 11 commercially available drug-like molecules from the ZINC database [60].

### 2.4. EBOV VP30 Inhibitors

The transcriptional activity of EBOV VP30 is regulated by a series of phosphorylation and dephosphorylation events facilitated by host kinases and phosphatases [151]. Modulating these enzymes could therefore alter the activity of VP30. For instance, serine-arginine protein kinase 1 (SRPK1) and SRPK2 phosphorylate Ser29 of VP30 to initiate EBOV primary transcription, and an SRPK1/SRPK2 inhibitor, such as SRPIN340, was able to downregulate the primary viral transcription [61]. On the contrary, the host protein phosphatase 1 (PP1) and protein phosphatase 2A-B56 (PP2A-B56) regulate VP30 dephosphorylation, which mediates the initiation of secondary viral transcription [62,152]. In one study, a PP2A-B56 inhibitor that interfered with VP30 dephosphorylation indeed suppressed the proliferation of EBOV [62]. In another study published by Ammosova et al., the synthetic small molecule C31, which binds to the catalytic subunit of PP1α, was also found to inhibit EBOV replication [63]. 

### 2.5. EBOV VP24 Inhibitors

EBOV VP24 is essential for viral RNP complex assembly [19] and also plays an important role in countering the IFN response [21]. It has been shown that VP24-specific PMOs can protect mice from lethal EBOV challenge [64]. Potential VP24 inhibitors have been identified through in silico screening, including cycloartocarpin [67]; ZINC000095486070; ZINC000003594643; ZINC000095486008; sarcophine from African natural product-derived compounds [68]; and the flavonoids gossypetin, taxifolin, and tricetin [65]. A recent study published by Fanunza et al. confirmed that the flavonoids gossypetin, taxifolin, and tricetin indeed blocked VP24′s anti-IFN function [66]. They also found that another flavonoid with a similar structure, quercetin, could directly interfere with VP24 binding to KPN-α to restore interferon-stimulated gene 15 (ISG15) transcription, thus blocking EBOV replication in vitro [66]. 

### 2.6. EBOV Polymerase Inhibitors

Viral polymerase, which plays an essential role in genome transcription and replication in host cells, represents another target for antiviral drug development. Efforts have been made to block EBOV replication by using stable nucleic acid-lipid particles (SNALPs) to encapsulate siRNAs. A cocktail of four different siRNAs targeting EBOV L has been shown to protect guinea pigs from lethal EBOV challenge [73]. Nucleoside analogs, such as brincidofovir, lamivudine, favipiravir, zidovudine, and galidesivir (BCX4430, immucillin A), have been proposed as potential candidates and investigated. Brincidofovir was tested in a phase 2 trial in Liberia in 2015, but the therapeutic effect was undetermined due to an early termination of the trial and a small sample size [153]. Lamivudine and zidovudine were proven ineffective in vitro [74]. Favipiravir rescued lethal EBOV challenge in mice [70,122], but provided low protection in NHP studies [123,124] as well as in clinical trials [154]. Galidesivir was able to inhibit EBOV minigenome replication [71] and protected NHP against lethal EBOV infection [125]. The drug also appeared to be safe and well tolerated in a phase I clinical trial [155]. On the other hand, remdesivir (GS-5734), a prodrug of an adenosine analogue, was designed to treat EBOV infection and has shown 100% protection from lethal disease in EBOV-infected rhesus monkeys [72]. Unfortunately, in the Pamoja Tulinde Maisha trial, the overall mortality of remdesivir-treated patients was 53.1%, which appeared higher than the ZMapp, MAb114, and REGN-EB3 groups; thus, the regimen was terminated along with ZMapp [146]. Finally, a computational study predicted pairs of approved nucleotide analogues (ribavirin + tenofovir, favipiravir + tenofovir, abacavir + tenofovir, or telbivudine + tenofovir) that may inhibit EBOV polymerase better in combination than when used individually [75]; however, their effectiveness remains to be validated. 

### 2.7. Host-Targeting Agents

Targeting essential host factors in the viral life cycle is another key strategy to perturb viral infection. Moreover, compared to viral proteins, host factors impose a higher barrier to mutations, making them potential candidates for antiviral intervention. 

Several host proteins are involved in EBOV entry. AMP-activated protein kinase (AMPK) is essential for EBOV macropinocytosis, and the AMPK inhibitor compound C is able to block EBOV GP-mediated infection of primary human macrophages [80]. Similarly, pyridinyl imidazole inhibitors of p38 mitogen-activated protein kinase (MAPK) were also found to inhibit EBOV macropinocytosis [81]. Host N-acetylglucosamine-1-phosphate transferase (GlcNAc-1-phosphotransferase) was identified as another potential EBOV antiviral target for its functions in lysosome transportation. The disruption of GlcNAc-1-phosphotransferase activity using PF-429242, an inhibitor of S1P (cellular proprotein convertase sterol regulatory element-binding protein (SREBP) site 1 protease) responsible for the cleavage of the GlcNAc-1-phosphotransferase precursor, was able to block EBOV entry, which requires its GP to be processed in the lysosome [82]. The anti-protozoal and emetic agent emetine was also shown to induce lysosomal dysfunction and inhibit EBOV entry [83]. In addition, ion channel inhibitors, such as amiodarone, dronedarone, and verapamil, were also found to inhibit EBOV entry [76], possibly by interfering with the viral membrane fusion to the endosomal membrane [77]. Although amiodarone was given as a compassionate therapy in Sierra Leone [156], its clinical trial was withdrawn and its effect was undetermined (NCT02307591). Further analyses suggested that the drugs’ anti-EBOV activity appeared questionable in mice and guinea pig models [78,79]. On the other hand, several G protein-coupled receptor (GPCR) antagonists were found to exhibit anti-EBOV activity, especially at the post-binding step [84], suggesting a potential role of GPCRs in EBOV entry. Several selective estrogen receptor modulators (SERMs) were found to inhibit EBOV entry by inducing endolysosomal calcium accumulation [85]. BIBX 1382, previously known as a ErbB kinase inhibitor, was also shown to block EBOV entry [86]. 

Host proteins that facilitate EBOV transcription and translation are another group of potential antiviral targets. Heat shock protein 90 (Hsp90) is an important host factor that is involved in protein folding, and the inhibition of Hsp90 with Geldanamycin, 17-AAG, and radicicol has been shown to reduce EBOV replication [88]. Another ER-resident, heat shock protein family A (Hsp70) member 5 (HspA5), is required for the production of EBOV transcripts and proteins, and inhibiting HspA5 with epigallocatechin gallate (EGCG) and HspA5-specific PMOs could inhibit Ebola replication in vitro and protect mice from lethal EBOV infection, respectively [87]. On the other hand, the inhibition of the host eIF4A translation initiation complex by silvestrol, a natural plant-derived compound isolated from *Aglaia foveolate*, was also shown to reduce EBOV replication in vitro [89]. A high-throughput screening using the minigenome system further identified potential EBOV replication inhibitors with a high selective index, including the protein synthesis inhibitors emetine and cycloheximide, and an inhibitor of inosine monophosphate dehydrogenase (IMPDH), mycophenolic acid [90].

Increasing host immune responses represents an additional strategy to combat viral infection. For example, targeting the cellular enzyme S-adenosyl-l-homocysteine hydrolase (SAH) with the adenosine analogues carbocyclic 3-deazaadenosine (Ca-c^3^ Ado) [91,126] or 3-deazaneplanocin A (c^3^-Npc A) [126,127] was shown to protect mice from lethal EBOV infection, possibly due to the antiviral immunity triggered by incompletely capped viral mRNA [157]. Another group found that the FDA-approved anti-protozoal drug nitazoxanide (NTZ) could broadly enhance innate antiviral immunity, which inhibited EBOV replication and counteracted EBOV VP35′s immune suppression [93]. On the other hand, type I and II IFNs have also been explored, but with mixed results. IFNs appeared to be weak inhibitors in cell-based EBOV infection assays [92]. When tested in vivo, while IFN-γ protected mice from lethal EBOV challenge [128], IFN-α [129] and IFN-β [130] did not provide any survival benefits in NHP despite a longer time to death. A retrospective study suggested that patients treated with support care only were 1.5–1.9 fold more likely to die than those who received IFN-β treatment [158]. 

The proteins involved in viral egress could also be targeted. For instance, TSG101 is a housekeeping protein that escorts proteins from the cytosol to the cell membrane, and viruses often hijack this protein to help release progeny viruses. The compound FGI-104 was identified as a TSG101 inhibitor that could interfere with the budding of several viruses and protected mice from lethal EBOV challenge [94]. 

Finally, as EBOV infection induces coagulation abnormalities, agents that correct such dysregulation have been studied. Some examples include the recombinant nematode anticoagulant protein c2 (rNAPc2), an inhibitor of tissue factor-initiated coagulation [95], and the recombinant human activated protein C (rhAPC), which regulates both coagulation and inflammation [96]. Both agents provided partial protection in rhesus macaques when given after lethal EBOV challenges (Table 2).

### 2.8. Combination Treatments

Drug combinations targeting multiple host or viral factors may prevent the development of viral resistance and reveal synergetic effects to control viral infection, with the benefit of lowering the individual drug dosage and toxicity. For instance, combinations of PMOs (targeting VP24, VP35, and L) [97], positively charged PMOs (PMOplus AVI-6002 targeting VP24, VP35, and L) [98], or SNALPs in a cocktail containing different siRNAs (also targeting VP24, VP35, and L) [131] have been reported, and the SNALP cocktail was shown to protect rhesus macaques from lethal EBOV challenge [131]. A combination of mAbs targeting different viral proteins (e.g., anti-GP and anti-VP40) [101] or combinations of IFNs and nucleoside analogs [102] have also been shown to inhibit the replication of EBOV VLP in vitro. In addition, another study showed that adenovirus-vectored IFN-α (Ad-IFN-α) was able to enhance the protection of adenovirus-based vaccine expressing EBOV GP (Ad-CAGoptZGP) in rodents [99] and ZMAb in NHP [100]. Other combinations of FDA-approved drugs have also been suggested. These include the three-drug combinations toremifene + mefloquine + posaconazole and toremifene + clarithromycin + posaconazole, which inhibit lysosomal calcium release, acid sphingomyelinase activity, and NPC-1 protein function [103]. Other synergistic drug pairs that target both the entry and post-entry steps of EBOV infection include aripiprazole + piperacetazine, sertraline + toremifene, sertraline + bepridil, amodiaquine + clomiphene [104], digitoxin + tetrandrine, digitoxin + tamoxifen, digitoxin + fluvastatin, and tamoxifen + fluvastatin [105].

## 3. Future Prospects

As reviewed above, several inhibitors are in different phases of preclinical development, with further validation studies being required for many candidates identified in vitro and in silico. Currently, most studies have focused on GP inhibitors and polymerase inhibitors, and neutralizing antibodies seem to produce the best outcomes in clinical settings; however, multifunctional EBOV proteins, such as VP35, VP40, VP30, and VP24, are also attractive targets and may be included in antiviral combinations. Despite their generally low barrier to mutations, the success of targeting viral factors, for example with the use of combination direct-acting antivirals (DAAs) to cure HCV infection and the multi-pronged highly active antiretroviral therapy (HAART) to control HIV infection, suggests that targeting multiple viral proteins could be an attractive strategy for antiviral development. Although EBOV was reportedly not undergoing rapid evolution in humans at least during the 2013–2015 period of the global outbreak [159], viral escape mutations have been observed in NHP treated with the MB-003 antibody cocktail [160]. Thus, a combination treatment approach, similar to DAA combination or HAART, could help to minimize escape mutants and may be considered for future therapeutic developments against EBOV infection. Several synergistic combinations of FDA-approved drugs have also been proposed but have not been investigated in vivo [103,104,105]. 

A number of other potential candidates have been suggested to inhibit EBOV entry in vitro, but their targets were not identified. These include 17 compounds from the Molecular Libraries Small Molecule Repository (MLSMR) library that targeted different steps of EBOV entry [106], the synthesized compound **8a** and its derivatives [107], the antibiotics azithromycin [78] and teicoplanin [108], the antimalarial chloroquine [78,109,110,132], several other FDA-approved compounds [110], and the flavonoid derivative quercetin 3-β-O-D-glucoside (Q3G) [112]. Q3G was shown to fully protect mice from lethal EBOV challenge [112], whereas azithromycin, teicoplanin, and chloroquine appeared ineffective and/or toxic at the doses tested in animal studies [54,78,111,132].

In terms of potential toxicity, FDA-approved drugs have better characterized profiles. For instance, digitoxin is known for its cardiotoxicity [161], and sertraline can cause liver injury and serotonin syndrome [162]. As for other newly developed antivirals including antibodies and compounds, their potential toxicity requires further investigation.

Finally, novel methods of drug development or drug delivery may also further improve anti-EBOV therapeutics. For example, bioinformatics or artificial intelligence may help facilitate drug discovery or design. Drug delivery systems, such as adeno-associated virus (AAV) vectors, have been shown to successfully transfer monoclonal antibodies genes into mice with a single injection and prophylactically protect them from EBOV challenge [115,116,117] (Table 2). AAV delivery can reduce the costs of large-quantity antibody production and repeated injections; the protective effect lasted for at least 5 months in mice [117]. These approaches could help fast-track drug development against EBOV and enhance global preparedness to manage current and potential future EBOV outbreaks. 

## Figures and Tables

**Table 1:** Potential antiviral candidates against EBOV infection.

Category	Drug(s)	Stage	IC_50_ (In Vitro)	Ref.
VP35 inhibitors	VP35 PMO and P-PMO	In vivo	0.9–1.25 μM (P-PMO; wtEBOV)	[22]
	Myricetin	In vitro	2.7 μg/mL (enzymatic)	[23]
	MCCB4	In vitro	4.8 μM (EBOV minigenome)	[24]
	Anti-VP35 scFvs	In vitro	N.D.	[25]
VP40 inhibitors	Anti-VP40 scFv	In vitro	N.D.	[26]
	Quinoxaline-based inhibitors	In vitro	N.D.	[27]
	Sorbitol, mannitol, galactitol	In silico	N.D.	[28]
	Pyrimidinediones class molecules	In silico	N.D.	[29]
GP inhibitors	mAb114	Licensed (Ebanga™)	0.09 μg/mL (wtEBOV)	[30]
	REGN-EB3 (REGN3470, REGN3471, REGN3479)	Licensed (Inmazeb™)	0.39 nM (EBOVpp)	[31]
	ZMapp^TM^ (c13C6, c2G4, c4G7)	Clinical trial	N.D.	[32]
	MIL77E (MIL77-1, MIL77-3)	In vivo	1–10, 10–100 μg/mL (EBOV-GFP)	[33]
	ZMAb (m1H3, m2G4, m4G7)	In vivo	18.75, 4.325, 0.678125 μg/mL (EBOVpp)	[34]
	MB-003 (c13C6, h13F6, c6D8)	In vivo	N.D.	[35,36,37,38]
	KZ52	In vivo	0.3–0.9 μg/mL (wtEBOV)	[39]
	Q206, Q314, Q411	In vivo	0.36, 0.78, 0.43 μg/mL (EBOVpp); 7.08, 42.96, 15.24 μg/mL (EBOV-GFP)	[40]
	2G1, 5E1, 5E9	In vivo	2.80, 11.13, 4.19 μg/mL (EBOV-GFP)	[41]
	6D6	In vivo	0.05–0.12 μg/mL (EBOVpp)	[42]
	m8C4	In vivo	1.5 μg/mL (EBOVpp)	[43]
	Bis-mAbs	In vivo	1.5–6.4 nM (EBOVpp); 0.5–1.1 nM (wtEBOV)	[44]
	CA45	In vivo	3.0–4.63 nM (EBOVpp); 28.3 nM (live virus)	[45]
	FVM04	In vivo	0.8–3.4 μg/mL (EBOVpp)	[46]
	040, 66-3-9C, 6662, 6541	In vivo	0.1–10 μg/mL (EBOVpp)	[47]
	MBP134^AF^	In vivo	0.1–1 nM (EBOVpp)	[48]
	rEBOV-520, rEBOV-548	In vivo	0.1–1, 1–10 μg/mL (EBOV-GFP)	[49]
	EBOV/SUDV pAb	In vivo	N.D.	[50]
	F(ab’)_2_	In vivo	1.4–1.7 μg/mL (EBOV-GFP)	[51]
	rhMBL	In vivo	N.D.	[52]
	Clomiphene	In vivo	0.755–2.42 μM (EBOV-GFP); 3.83–11.1 μM (wtEBOV)	[53]
	Toremifene	In vivo	0.0255–0.162 μM (EBOV-GFP); 0.973–1.73 μM (wtEBOV)	[53]
	Bepridil	In vivo	3.21–5.08 μM (EBOV-GFP); 4.54 μM (wt EBOV)	[54]
	Sertraline	In vivo	1.44–3.13 μM (EBOV-GFP); 3.73–8.62 μM (wtEBOV)	[54]
	HP-HSA	In vitro	0.068 μM (EBOVpp)	[55]
	PPCM	In vitro	N.D.	[56]
	Sclareol, sclareolide	In vitro	2.4, 8.0 μM (EBOVpp)	[57]
	Compound 7	In vitro	10 μM (EBOV-GFP)	[58]
	Compound 118, compound 118a	In vitro	3.1, 0.05 μM (EBOVpp)	[59]
	11 compounds from ZINC database	In vitro	1.79–36.66 μM (EBOVpp)	[60]
VP30 inhibitors	SRPK1/SRPK2 inhibitor (SRPIN340)	In vitro	N.D.	[61]
	PP2A-B56 inhibitor	In vitro	N.D.	[62]
	PP1α inhibitor (C31)	In vitro	N.D.	[63]
VP24 inhibitors	VP24 PMOs	In vivo	8–11 nM (enzymatic)	[64]
	Gossypetin, taxifolin, tricetin	In vitro	N.D.	[65,66]
	Quercetin	In vitro	7.4 μM (enzymatic)	[66]
	Cycloartocarpin	In silico	N.D.	[67]
	ZINC000095486070, ZINC000003594643, ZINC000095486008, Sarcophine	In silico	N.D.	[68]
Polymerase inhibitors	Brincidofovir	Clinical trial	120 nM–1.3 µM	[69]
	Favipiravir	Clinical trial	67 μM (wtEBOV)	[70]
	Galidesivir	Clinical trial	11.8 μM (wtEBOV)	[71]
	Remdesivir	Clinical trial	0.06–0.14 μM (EBOV-GFP or wtEBOV)	[72]
	SNALPs (L)	In vivo	N.D.	[73]
	Lamivudine, zidovudine	In vitro	>320 µM (wtEBOV)	[74]
	Pairs of approved nucleotide analogues	In silico	N.D.	[75]
Host-targeting inhibitors—Viral entry	Amiodarone	Clinical trial	2.02 μM (EBOVpp); 0.25 μg/mL (wtEBOV)	[76]
	Amiodarone	Clinical trial	5.6 μM (EBOVpp)	[77]
	Amiodarone	Clinical trial	0.81 μM (EBOVpp); 7.6 μM (EBOV-GFP)	[78]
	Amiodarone	Clinical trial	5.5–15.9 μM (wtEBOV)	[79]
	AMPK inhibitor (Compound C)	In vitro	∼6 μM (EBOVpp)	[80]
	MAPK inhibitors	In vitro	2.67–8.26 μM (EBOV-GFP)	[81]
	GlcNAc-1-phosphotransferase inhibitor (PF-429242)	In vitro	0.80 μM (EBOVpp); 0.95 μM (EBOV-ZsG)	[82]
	Emetine	In vitro	10.2 μM (EBOV VLP); 16.9 nM (EBOV-GFP)	[83]
	Dronedarone, verapamil	In vitro	N.D.	[76]
	GPCR antagonists	In vitro	3.7–19.4 μM (EBOV-GFP)	[84]
	SERMs	In vitro	N.D.	[85]
	ErbB kinase inhibitor (BIBX 1382)	In vitro	1.1 μM (EBOV-GFP)	[86]
Host-targeting inhibitors—Viral replication	HspA5 inhibitors	In vivo	50–60 μM (EGCG; live virus)	[87]
	Hsp90 inhibitors	In vitro	43.8–394.5 nM (EBOV-GFP)	[88]
	eIF4A inhibitor (silvestrol)	In vitro	∼0.8 nM (live virus)	[89]
	Emetine, cycloheximide, mycophenolic acid	In vitro	1.474, 0.608, 0.316 μM (EBOV-GFP)	[90]
Host-targeting inhibitors—Antiviral immunity	SAH inhibitor (Ca-c^3^ Ado)	In vivo	30 μM (wtEBOV)	[91]
	SAH inhibitor (c^3^-Npc A)	In vivo	2 μM (wtEBOV)	[91]
	IFNs	In vivo	<10–5102 U/mL (wtEBOV)	[92]
	Nitazoxanide	In vitro	N.D.	[93]
Host-targeting inhibitors—Viral egress	TSG101 inhibitor (FGI-104)	In vivo	10 μM (EBOV-GFP)	[94]
Host-targeting inhibitors—Anticoagulant	rNAPc2	In vivo	N.D.	[95]
	rhAPC	In vivo	N.D.	[96]
Combination Treatments	PMOs (VP24, VP35, L)	In vivo	N.D.	[97]
	PMOplus AVI-6002 (VP24, VP35, L)	In vivo	N.D.	[98]
	Ad-IFN-α + Ad-CAGoptZGP	In vivo	N.D.	[99]
	Ad-IFN-α + ZMAb	In vivo	N.D.	[100]
	anti-GP + anti-VP40	In vitro	N.D.	[101]
	Combinations of IFNs and nucleoside analogs	In vitro	N.D.	[102]
	Toremifene + mefloquine + posaconazole, toremifene + clarithromycin + posaconazole	In vitro	1.08, 0.97 μM (Ebola VLP)	[103]
	Aripiprazole + piperacetazine, sertraline + toremifene, sertraline + bepridil, amodiaquine + clomiphene	In vitro	N.D.	[104]
	Digitoxin + tetrandrine, digitoxin + tamoxifen, digitoxin + fluvastatin, tamoxifen + fluvastatin	In vitro	N.D.	[105]
Inhibitors with unknown target(s)	17 compounds from MLSMR library	In vitro	1.6–25.6 μM (EBOV-GFP)	[106]
	Compound 8a and derivatives	In vitro	2.5–30 μM (EBOVpp)	[107]
	Azithromycin	In vivo	1.3 μM (EBOVpp); 5.1 μM (EBOV-GFP)	[78]
	Teicoplanin	In vivo	2.38 μM (EBOVpp)	[108]
	Teicoplanin	In vivo	7.28 μM (EBOV-GFP)	[54]
	Chloroquine	In vivo	4.7 μM (EBOVpp); 16 μM (EBOV-GFP)	[78]
	Chloroquine	In vivo	3.319 μM (EBOVpp)	[109]
	Chloroquine	In vivo	15.3 μM (EBOV VLP)	[110]
	Chloroquine	In vivo	4.7 μM (EBOVpp); 16 μM (EBOV-GFP)	[78]
	Chloroquine	In vivo	1.77 μg/mL (EBOV-GFP)	[111]
	Quercetin 3-β-O-D-glucoside	In vivo	5.3 μM (EBOV-GFP)	[112]
	FDA-approved compounds	In vitro	<25 μM (EBOV VLP)	[110]

AMPK, AMP-activated protein kinase; EBOV, Ebola virus; EBOV-GFP, EBOV expressing green fluorescence protein; EBOVpp, EBOV pseudoparticle; GlcNAc, N-acetylglucosamine; GPCR, G protein-coupled receptor; HP-HSA, 3-hydroxyphthalic anhydride-modified human serum albumin; Hsp, heat shock protein; IC_50_, 50% inhibitory concentration; IFN, interferon; mAb, monoclonal antibody; MAPK, mitogen-activated protein kinase; N.D., not determined; pAb, polyclonal antibody; PMO, phosphorodiamidate morpholino oligomers; P-PMO, PMO conjugated to cell-penetrating peptide; PPCM, polyphenylene carboxymethylene; PP1, protein phosphatase 1; PP2A-B56, protein phosphatase 2A-B56; rhAPC, recombinant human activated protein C; rhMBL, recombinant human mannose-binding lectin; rNAPc2, recombinant nematode anticoagulant protein c2; SAH, S-adenosyl-l-homocysteine hydrolase; scFv, single-chain variable fragment; SERM, selective estrogen receptor modulator; SNALP, stable nucleic acid-lipid particle; SRPK, serine-arginine protein kinase; SUDV, Sudan virus; VLP, virus-like particle; VP, viral protein; wtEBOV, wild-type EBOV.

**Table 2:** Animal studies of EBOV antiviral therapeutics.

Category	Drug(s)	Animal Model	Treatment Time Post-Infection; Dose	Best Survival	Ref.
VP35 inhibitor	VP35 PMO	Mouse	−24 h, −4 h; 500 μg	85%	[22]
	VP35 P-PMO	Mouse	−24 h, −4 h; 500 μg	100%	[22]
	VP35 P-PMO	Mouse	D1; 500 μg	100%	[22]
GP inhibitors	mAb114	Rhesus macaque	D5-7; 50 mg/kg	100%	[30]
	MIL77E	Rhesus macaque	D3, 6, 9; 50 mg/kg	100%	[33]
	ch133 + ch226	Rhesus macaque	D(−1), 1, 3; 25 mg/kg/mAb	33%	[113]
	ZMAb	Guinea pig	D2; 2 mg 4G7 + 1.5 mg 1H3 + 1.5 mg 2G4	100%	[34]
	ZMAb	Cynomolgus macaque	D1, 4, 7; 25 mg/kg	100%	[114]
	ZMAb	Cynomolgus macaque	D2, 5, 8; 25 mg/kg	50%	[114]
	MB-003	Rhesus macaque	D2, 6, 8, 10; 16.7 mg/kg/mAb	67%	[37]
	MB-003	Rhesus macaque	Three doses initiated after symptom onset (by 120 h.p.i.); 16.7 mg/kg/mAb	43%	[38]
	ZMapp^TM^	Rhesus macaque	D5, 8, 11; 50 mg/kg	100%	[32]
	AAV9-ZMapp	Mouse	D(−14); 3 × 10^11^ genome copies	>80%	[115]
	AAV9-ZMapp	Mouse	D(−21); 3 × 10^11^ genome copies	60–90%	[116]
	AAV9-c2G4	Mouse	D(−21); 3 × 10^11^ genome copies	90–100%	[116]
	AAV6.2FF-2G4 + AAV6.2FF-5D2	Mouse	D(−7) or (−14) or (−140); 4 × 10^11^ genome copies	100%	[117]
	REGN-EB3	Rhesus macaque	D5; 150 mg/kg	89%	[31]
	REGN-EB3	Rhesus macaque	D5, 8, 11; 50 mg/kg	100%	[31]
	KZ52	Guinea pig	−1 h or +1 h; 25 mg/kg	80–100%	[39]
	KZ52	Rhesus macaque	D1, 4; 50 mg/kg	0%	[118]
	Q206	Mouse	D2; 100 μg	66.6%	[40]
	Q314	Mouse	D1 or 2; 100 μg	33.3	[40]
	Q411	Mouse	D1; 100 μg	50.0	[40]
	2G1	Mouse	D1; 100 μg	100%	[41]
	5E1	Mouse	D1; 100 μg	100%	[41]
	5E9	Mouse	D1; 100 μg	100%	[41]
	6D6	Mouse	D1; 100 μg	100%	[42]
	m8C4	Mouse	+2 h, D3; 25 mg/kg	47%	[43]
	Bis-mAbs	Mouse	D1; 200 μg	70–100%	[44]
	040 + 66-3-9C + 6662 + 6541	Guinea pig	D3; 10 mg/kg/mAb	100%	[47]
	FVM04 + CA45	Guinea pig	D3; 2.5 mg/mAb	100%	[45]
	FVM04 + CA45	Guinea pig	D3; 1.25 mg/mAb	100%	[119]
	FVM04 + CA45	Rhesus macaque	D4; 20 mg/kg/mAb	100%	[119]
	ADI-15742	Mouse	D2; 300 μg	100%	[48]
	ADI-15878	Mouse	D2; 300 μg	80%	[48]
	MBP134^AF^	Guinea pig	D3; 3.3 mg	100%	[120]
	MBP134^AF^	Ferret	D2, 5 or D3, 6; 15 mg	100%	[121]
	MBP134^AF^	Rhesus macaque	D4; 25 mg/kg	100%	[121]
	rEBOV-520 + rEBOV-548	Rhesus macaque	D3, 6; 30 mg/kg	100%	[49]
	EBOV/SUDV pAb	Mouse	D1; 100 mg/kg	80%	[50]
	F(ab’)_2_	Rhesus macaque	D3-7, 9, 11; 100 mg/kg	100%	[51]
	F(ab’)_2_	Rhesus macaque	D5-9, 11, 13; 100 mg/kg	100%	[51]
	Clomiphene	Mouse	+1 h, D1, 3, 5, 7, 9; 60 mg/kg	90%	[53]
	Toremifene	Mouse	+1 h, D1, 3, 5, 7, 9; 60 mg/kg	50%	[53]
	Bepridil	Mouse	BID since +1 h for 10 days; 12 mg/kg	100%	[54]
	Sertraline	Mouse	BID since +1 h for 10 days; 10 mg/kg	70%	[54]
	rhMBL	Mouse	D0-10 (Q12H since 12 h.p.i.); 20 mg/kg	>40%	[52]
VP24 inhibitor	PMOs	Mouse	−24 h, −4 h; 1–50 μg	30–100%	[64]
RdRp inhibitors	SNALPs (L)	Guinea pig	+1 h, D1-6; 0.75 mg/kg	100%	[73]
	Favipiravir	Mouse	D6-13; 300 mg/(kg × d)	100%	[70]
	Favipiravir	Mouse	BID since +1 h for 14 days; 150 mg/kg	100%	[122]
	Favipiravir	Cynomolgus macaque	BID since D(−2) for 14 days; 180 mg/kg (LD: 250 mg/kg)	60%	[123]
	Favipiravir	Cynomolgus macaque	D(−3)-10; 200 mg/kg (LD: 400 mg/kg)	17%	[124]
	Favipiravir	Cynomolgus macaque	BID since +0.5–2 h for 14 days; 150 mg/kg (LD: 250 mg/kg)	0%	[124]
	Galidesivir	Mouse	BID; 150 mg/kg	>80%	[71]
	Galidesivir	Rhesus macaque	BID since D2 for 11 days; 25 mg/kg (LD: 100 mg/kg)	100%	[125]
	Galidesivir	Rhesus macaque	BID since D3 for 11 days; 25 mg/kg (LD: 100 mg/kg)	67%	[125]
	Remdesivir	Rhesus macaque	D3-14; 3 or 10 mg/kg (LD: 10 mg/kg)	100%	[72]
Host-targeting inhibitors	Amiodarone	Mouse	D0-7 BID; 90 mg/kg	10–40%	[78]
	Amiodarone	Guinea pig	Starting from D(−3); 160 mg/kg	0%	[79]
	HspA5 PMO	Mouse	D(−4), (−1), +1, +3; 7.5 mg/kg	100%	[87]
	SAH inhibitor (Ca-c^3^ Ado)	Mouse	Q8H since −24 h for 9 days; 0.7 mg/kg	100%	[91]
	SAH inhibitor (Ca-c^3^ Ado)	Mouse	D2; 80 mg/kg	100%	[126]
	SAH inhibitor (c^3^-Npc A)	Mouse	D4; 1 mg/kg	100%	[126,127]
	IFN-γ	Mouse	+6 h; 3.3μg	100%	[128]
	IFN-α	Cynomolgus macaque	Daily since +18 h; 2 × 10^7^ IU/kg	0%	[129]
	IFN-β	Rhesus Macaque	+18 h, D1, 3, 5, 7, 9; 10.5 µg/kg	0%	[130]
	TSG101 inhibitor (FGI-104)	Mouse	−2 h; D1-10; 10 mg/kg	100%	[94]
	rNAPc2	Rhesus macaque	D1-14; 30 μg/kg	33%	[95]
	rhAPC	Rhesus macaque	Continuous infusion since 1 h.p.i.; 2 mg/m^2^/h	18%	[96]
Combination Treatments	PMOs (VP24, VP35, L)	Mouse	D1; 500 μg	100%	[97]
	PMOs (VP24, VP35, L)	Guinea pig	D4; 30 mg	67%	[97]
	PMOs (VP24, VP35, L)	Rhesus macaque	D(−2), 0–9; 12.5–100 mg	50%	[97]
	PMOplus AVI-6002 (VP24, VP35, L)	Rhesus macaque	+0.5–1 h, D1-14; 28 or 40 mg/kg	60%	[98]
	SNALPs (L, VP35, and VP24)	Rhesus macaque	+0.5 h, D1-6; 2 mg/kg	100%	[131]
	Ad-IFN-α + Ad-CAGoptZGP	Guinea pig	+0.5 h; Ad-IFN-α 2 × 10^8^ infectious particles + Ad-CAGoptZGP 10^10^ infectious particles	100%	[99]
	Ad-IFN-α + ZMAb	Cynomolgus macaque	D3; Ad-IFN 10^9^ PFU/kg + ZMAb 50 mg/kgD6, 9; ZMAb 50 mg/kg	100%	[100]
Inhibitors with unknown target(s)	Azithromycin	Mouse	D0-7 BID; 100 mg/kg	10–60%	[78]
	Azithromycin	Guinea pig	D0-7; 6 mg/kg	10%	[78]
	Teicoplanin	Mouse	+1 h, D1-9; 14 mg/kg	0%	[54]
	Chloroquine	Mouse	D0-7 BID; 90 mg/kg	70–80%	[78]
	Chloroquine	Guinea pig	D0-7; 25 mg/kg	0%	[78]
	Chloroquine	Guinea pig	BID since +6 h; 33.75 mg/kg	0%	[132]
	Chloroquine	Hamster	50 mg/kg	0%	[111]
	Quercetin 3-β-O-D-glucoside	Mouse	−0.5 h, D2, 4, 6, 8, 10; 50 mg/kg	100%	[112]
	Quercetin 3-β-O-D-glucoside	Mouse	D1, 3, 5, 7, 9, 11; 50 mg/kg	30%	[112]

BID, twice a day; D, day; h, hour; LD, loading dose.

## References

[1] Kuhn J.H., Amarasinghe G.K., Perry D.L., Howley P.M., Knipe D.M., Whelan S. (2021). Filoviridae. Fields Virology.

[2] Kuhn J.H., Amarasinghe G.K., Basler C.F., Bavari S., Bukreyev A., Chandran K., Crozier I., Dolnik O., Dye J.M., Formenty P.B.H. (2019). ICTV Virus Taxonomy Profile: Filoviridae. J. Gen. Virol..

[3] Jacob S.T., Crozier I., Fischer W.A., Hewlett A., Kraft C.S., Vega M.-A.d.L., Soka M.J., Wahl V., Griffiths A., Bollinger L. (2020). Ebola virus disease. Nat. Rev. Dis. Primers.

[4] Dudas G., Carvalho L.M., Bedford T., Tatem A.J., Baele G., Faria N.R., Park D.J., Ladner J.T., Arias A., Asogun D. (2017). Virus genomes reveal factors that spread and sustained the Ebola epidemic. Nature.

[5] Dietzel E., Schudt G., Krähling V., Matrosovich M., Becker S. (2017). Functional characterization of adaptive mutations during the West African Ebola virus outbreak. J. Virol..

[6] World Health Organization Ebola Virus Disease. https://www.who.int/health-topics/ebola/#tab=tab_1.

[7] U.S. Centers for Disease Control and Prevention Ebola (Ebola Virus Disease). https://www.cdc.gov/vhf/ebola/index.html.

[8] Mate S.E., Kugelman J.R., Nyenswah T.G., Ladner J.T., Wiley M.R., Cordier-Lassalle T., Christie A., Schroth G.P., Gross S.M., Davies-Wayne G.J. (2015). Molecular Evidence of Sexual Transmission of Ebola Virus. N. Engl. J. Med..

[9] European Medicines Agency First Vaccine to Protect against Ebola. https://www.ema.europa.eu/en/news/first-vaccine-protect-against-ebola.

[10] U.S. Food and Drug Administration First FDA-Approved Vaccine for the Prevention of Ebola Virus Disease, Marking a Critical Milestone in Public Health Preparedness and Response. https://www.fda.gov/news-events/press-announcements/first-fda-approved-vaccine-prevention-ebola-virus-disease-marking-critical-milestone-public-health.

[11] World Health Organization Four Countries in the African Region License Vaccine in Milestone for Ebola Prevention. https://www.who.int/news/item/14-02-2020-four-countries-in-the-african-region-license-vaccine-in-milestone-for-ebola-prevention.

[12] Woolsey C., Geisbert T.W. (2021). Current state of Ebola virus vaccines: A snapshot. PLoS Pathog..

[13] U.S. Food and Drug Administration FDA Approves First Treatment for Ebola Virus. https://www.fda.gov/news-events/press-announcements/fda-approves-first-treatment-ebola-virus.

[14] U.S. Food and Drug Administration FDA Approves Treatment for Ebola Virus. https://www.fda.gov/drugs/news-events-human-drugs/fda-approves-treatment-ebola-virus.

[15] Côté M., Misasi J., Ren T., Bruchez A., Lee K., Filone C.M., Hensley L., Li Q., Ory D., Chandran K. (2011). Small molecule inhibitors reveal Niemann–Pick C1 is essential for Ebola virus infection. Nature.

[16] Falasca L., Agrati C., Petrosillo N., Di Caro A., Capobianchi M., Ippolito G., Piacentini M. (2015). Molecular mechanisms of Ebola virus pathogenesis: Focus on cell death. Cell Death. Differ..

[17] Martínez M.J., Biedenkopf N., Volchkova V., Hartlieb B., Alazard-Dany N., Reynard O., Becker S., Volchkov V. (2008). Role of Ebola virus VP30 in transcription reinitiation. J. Virol..

[18] Mühlberger E., Weik M., Volchkov V.E., Klenk H.-D., Becker S. (1999). Comparison of the transcription and replication strategies of Marburg virus and Ebola virus by using artificial replication systems. J. Virol..

[19] Banadyga L., Hoenen T., Ambroggio X., Dunham E., Groseth A., Ebihara H. (2017). Ebola virus VP24 interacts with NP to facilitate nucleocapsid assembly and genome packaging. Sci. Rep..

[20] Cárdenas W.B., Loo Y.-M., Gale M., Hartman A.L., Kimberlin C.R., Martínez-Sobrido L., Saphire E.O., Basler C.F. (2006). Ebola virus VP35 protein binds double-stranded RNA and inhibits alpha/beta interferon production induced by RIG-I signaling. J. Virol..

[21] Mateo M., Leung L.W., Basler C.F., Volchkov V.E. (2010). Ebolavirus VP24 binding to karyopherins is required for inhibition of interferon signaling. J. Virol..

[22] Enterlein S., Warfield K.L., Swenson D.L., Stein D.A., Smith J.L., Gamble C.S., Kroeker A.D., Iversen P.L., Bavari S., Muhlberger E. (2006). VP35 knockdown inhibits Ebola virus amplification and protects against lethal infection in mice. Antimicrob. Agents Chemother..

[23] Daino G.L., Frau A., Sanna C., Rigano D., Distinto S., Madau V., Esposito F., Fanunza E., Bianco G., Taglialatela-Scafati O. (2018). Identification of Myricetin as an Ebola Virus VP35–Double-Stranded RNA Interaction Inhibitor through a Novel Fluorescence-Based Assay. Biochemistry.

[24] Easton V., McPhillie M., Garcia-Dorival I., Barr J.N., Edwards T.A., Foster R., Fishwick C., Harris M. (2018). Identification of a small molecule inhibitor of Ebola virus genome replication and transcription using in silico screening. Antivir. Res..

[25] Flego M., Frau A., Accardi L., Mallano A., Ascione A., Gellini M., Fanunza E., Vella S., Di Bonito P., Tramontano E. (2019). Intracellular human antibody fragments recognizing the VP35 protein of Zaire Ebola filovirus inhibit the protein activity. BMC Biotechnol..

[26] Teimoori S., Seesuay W., Jittavisutthikul S., Chaisri U., Sookrung N., Densumite J., Saelim N., Chulanetra M., Maneewatch S., Chaicumpa W. (2016). Human transbodies to VP40 inhibit cellular egress of Ebola virus-like particles. Biochem. Biophys. Res. Commun..

[27] Loughran H.M., Han Z., Wrobel J.E., Decker S.E., Ruthel G., Freedman B.D., Harty R.N., Reitz A.B. (2016). Quinoxaline-based inhibitors of Ebola and Marburg VP40 egress. Bioorganic Med. Chem. Lett..

[28] Nagarajan N., Yapp E.K., Le N.Q.K., Yeh H.-Y. (2019). In silico screening of sugar alcohol compounds to inhibit viral matrix protein VP40 of Ebola virus. Mol. Biol. Rep..

[29] El-Din H.M.A., Loutfy S.A., Fathy N., Elberry M.H., Mayla A.M., Kassem S., Naqvi A. (2016). Molecular docking based screening of compounds against VP40 from Ebola virus. Bioinformation.

[30] Corti D., Misasi J., Mulangu S., Stanley D.A., Kanekiyo M., Wollen S., Ploquin A., Doria-Rose N.A., Staupe R.P., Bailey M. (2016). Protective monotherapy against lethal Ebola virus infection by a potently neutralizing antibody. Science.

[31] Pascal K.E., Dudgeon D., Trefry J.C., Anantpadma M., Sakurai Y., Murin C.D., Turner H.L., Fairhurst J., Torres M., Rafique A. (2018). Development of Clinical-Stage Human Monoclonal Antibodies That Treat Advanced Ebola Virus Disease in Nonhuman Primates. J. Infect. Dis..

[32] Qiu X., Wong G., Audet J., Bello A., Fernando L., Alimonti J.B., Fausther-Bovendo H., Wei H., Aviles J., Hiatt E. (2014). Reversion of advanced Ebola virus disease in nonhuman primates with ZMapp. Nature.

[33] Qiu X., Audet J., Lv M., He S., Wong G., Wei H., Luo L., Fernando L., Kroeker A., Fausther Bovendo H. (2016). Two-mAb cocktail protects macaques against the Makona variant of Ebola virus. Sci. Transl. Med..

[34] Qiu X., Fernando L., Melito P.L., Audet J., Feldmann H., Kobinger G., Alimonti J.B., Jones S.M. (2012). Ebola GP-specific monoclonal antibodies protect mice and guinea pigs from lethal Ebola virus infection. PLoS Negl. Trop. Dis..

[35] Wilson J.A., Hevey M., Bakken R., Guest S., Bray M., Schmaljohn A.L., Hart M.K. (2000). Epitopes involved in antibody-mediated protection from Ebola virus. Science.

[36] Zeitlin L., Pettitt J., Scully C., Bohorova N., Kim D., Pauly M., Hiatt A., Ngo L., Steinkellner H., Whaley K.J. (2011). Enhanced potency of a fucose-free monoclonal antibody being developed as an Ebola virus immunoprotectant. Proc. Natl. Acad. Sci. USA.

[37] Olinger G.G., Pettitt J., Kim D., Working C., Bohorov O., Bratcher B., Hiatt E., Hume S.D., Johnson A.K., Morton J. (2012). Delayed treatment of Ebola virus infection with plant-derived monoclonal antibodies provides protection in rhesus macaques. Proc. Natl. Acad. Sci. USA.

[38] Pettitt J., Zeitlin L., Kim D.H., Working C., Johnson J.C., Bohorov O., Bratcher B., Hiatt E., Hume S.D., Johnson A.K. (2013). Therapeutic intervention of Ebola virus infection in rhesus macaques with the MB-003 monoclonal antibody cocktail. Sci. Transl. Med..

[39] Parren P.W., Geisbert T.W., Maruyama T., Jahrling P.B., Burton D.R. (2002). Pre- and postexposure prophylaxis of Ebola virus infection in an animal model by passive transfer of a neutralizing human antibody. J. Virol..

[40] Zhang Q., Gui M., Niu X., He S., Wang R., Feng Y., Kroeker A., Zuo Y., Wang H., Wang Y. (2016). Potent neutralizing monoclonal antibodies against Ebola virus infection. Sci. Rep..

[41] Fan P., Chi X., Liu G., Zhang G., Chen Z., Liu Y., Fang T., Li J., Banadyga L., He S. (2020). Potent neutralizing monoclonal antibodies against Ebola virus isolated from vaccinated donors. MAbs.

[42] Furuyama W., Marzi A., Nanbo A., Haddock E., Maruyama J., Miyamoto H., Igarashi M., Yoshida R., Noyori O., Feldmann H. (2016). Discovery of an antibody for pan-ebolavirus therapy. Sci. Rep..

[43] Holtsberg F.W., Shulenin S., Vu H., Howell K.A., Patel S.J., Gunn B., Karim M., Lai J.R., Frei J.C., Nyakatura E.K. (2016). Pan-ebolavirus and Pan-filovirus Mouse Monoclonal Antibodies: Protection against Ebola and Sudan Viruses. J. Virol..

[44] Frei J.C., Nyakatura E.K., Zak S.E., Bakken R.R., Chandran K., Dye J.M., Lai J.R. (2016). Bispecific Antibody Affords Complete Post-Exposure Protection of Mice from Both Ebola (Zaire) and Sudan Viruses. Sci. Rep..

[45] Zhao X., Howell K.A., He S., Brannan J.M., Wec A.Z., Davidson E., Turner H.L., Chiang C.I., Lei L., Fels J.M. (2017). Immunization-Elicited Broadly Protective Antibody Reveals Ebolavirus Fusion Loop as a Site of Vulnerability. Cell.

[46] Howell K.A., Qiu X., Brannan J.M., Bryan C., Davidson E., Holtsberg F.W., Wec A.Z., Shulenin S., Biggins J.E., Douglas R. (2016). Antibody Treatment of Ebola and Sudan Virus Infection via a Uniquely Exposed Epitope within the Glycoprotein Receptor-Binding Site. Cell Rep..

[47] Rijal P., Elias S.C., Machado S.R., Xiao J., Schimanski L., O’Dowd V., Baker T., Barry E., Mendelsohn S.C., Cherry C.J. (2019). Therapeutic Monoclonal Antibodies for Ebola Virus Infection Derived from Vaccinated Humans. Cell Rep..

[48] Wec A.Z., Herbert A.S., Murin C.D., Nyakatura E.K., Abelson D.M., Fels J.M., He S., James R.M., de La Vega M.-A., Zhu W. (2017). Antibodies from a Human Survivor Define Sites of Vulnerability for Broad Protection against Ebolaviruses. Cell.

[49] Gilchuk P., Murin C.D., Milligan J.C., Cross R.W., Mire C.E., Ilinykh P.A., Huang K., Kuzmina N., Altman P.X., Hui S. (2020). Analysis of a Therapeutic Antibody Cocktail Reveals Determinants for Cooperative and Broad Ebolavirus Neutralization. Immunity.

[50] Bounds C.E., Kwilas S.A., Kuehne A.I., Brannan J.M., Bakken R.R., Dye J.M., Hooper J.W., Dupuy L.C., Ellefsen B., Hannaman D. (2015). Human Polyclonal Antibodies Produced through DNA Vaccination of Transchromosomal Cattle Provide Mice with Post-Exposure Protection against Lethal Zaire and Sudan Ebolaviruses. PLoS ONE.

[51] Wang H., Wong G., Zhu W., He S., Zhao Y., Yan F., Rahim M.N., Bi Y., Zhang Z., Cheng K. (2019). Equine-origin immunoglobulin fragments protect nonhuman primates from Ebola virus disease. J. Virol..

[52] Michelow I.C., Lear C., Scully C., Prugar L.I., Longley C.B., Yantosca L.M., Ji X., Karpel M., Brudner M., Takahashi K. (2011). High-Dose Mannose-Binding Lectin Therapy for Ebola Virus Infection. J. Infect. Dis..

[53] Johansen L.M., Brannan J.M., Delos S.E., Shoemaker C.J., Stossel A., Lear C., Hoffstrom B.G., Dewald L.E., Schornberg K.L., Scully C. (2013). FDA-approved selective estrogen receptor modulators inhibit Ebola virus infection. Sci. Transl. Med..

[54] Johansen L.M., DeWald L.E., Shoemaker C.J., Hoffstrom B.G., Lear-Rooney C.M., Stossel A., Nelson E., Delos S.E., Simmons J.A., Grenier J.M. (2015). A screen of approved drugs and molecular probes identifies therapeutics with anti-Ebola virus activity. Sci. Transl. Med..

[55] Li H., Yu F., Xia S., Yu Y., Wang Q., Lv M., Wang Y., Jiang S., Lu L. (2017). Chemically modified human serum albumin potently blocks entry of Ebola pseudoviruses and viruslike particles. Antimicrob. Agents Chemother..

[56] Escaffre O., Juelich T.L., Freiberg A.N. (2019). Polyphenylene carboxymethylene (PPCM) in vitro antiviral efficacy against Ebola virus in the context of a sexually transmitted infection. Antivir. Res..

[57] Chen Q., Tang K., Guo Y. (2020). Discovery of sclareol and sclareolide as filovirus entry inhibitors. J. Asian Nat. Prod. Res..

[58] Basu A., Li B., Mills D.M., Panchal R.G., Cardinale S.C., Butler M.M., Peet N.P., Majgier-Baranowska H., Williams J.D., Patel I. (2011). Identification of a small-molecule entry inhibitor for filoviruses. J. Virol..

[59] Shaikh F., Zhao Y., Alvarez L., Iliopoulou M., Lohans C., Schofield C.J., Padilla-Parra S., Siu S.W., Fry E.E., Ren J. (2019). Structure-Based in silico screening identifies a potent ebolavirus inhibitor from a traditional chinese medicine library. J. Med. Chem..

[60] Singleton C.D., Humby M.S., Yi H.A., Rizzo R.C., Jacobs A. (2019). Identification of Ebola Virus Inhibitors Targeting GP2 Using Principles of Molecular Mimicry. J. Virol..

[61] Takamatsu Y., Krähling V., Kolesnikova L., Halwe S., Lier C., Baumeister S., Noda T., Biedenkopf N., Becker S., Whelan S.P.J. (2020). Serine-Arginine Protein Kinase 1 Regulates Ebola Virus Transcription. mBio.

[62] Kruse T., Biedenkopf N., Hertz E.P.T., Dietzel E., Stalmann G., López-Méndez B., Davey N.E., Nilsson J., Becker S. (2018). The Ebola virus nucleoprotein recruits the host PP2A-B56 phosphatase to activate transcriptional support activity of VP30. Mol. Cell.

[63] Ammosova T., Pietzsch C.A., Saygideğer Y., Ilatovsky A., Lin X., Ivanov A., Kumari N., Jerebtsova M., Kulkarni A., Petukhov M. (2018). Protein Phosphatase 1–Targeting Small-Molecule C31 Inhibits Ebola Virus Replication. J. Infect. Dis..

[64] Swenson D.L., Warfield K.L., Warren T.K., Lovejoy C., Hassinger J.N., Ruthel G., Blouch R.E., Moulton H.M., Weller D.D., Iversen P.L. (2009). Chemical modifications of antisense morpholino oligomers enhance their efficacy against Ebola virus infection. Antimicrob. Agents Chemother..

[65] Raj U., Varadwaj P.K. (2016). Flavonoids as Multi-target Inhibitors for Proteins Associated with Ebola Virus: In Silico Discovery Using Virtual Screening and Molecular Docking Studies. Interdiscip. Sci. Comput. Life Sci..

[66] Fanunza E., Iampietro M., Distinto S., Corona A., Quartu M., Maccioni E., Horvat B., Tramontano E. (2020). Quercetin Blocks Ebola Virus Infection by Counteracting the VP24 Interferon-Inhibitory Function. Antimicrob. Agents Chemother..

[67] Tambunan U., Nasution M. (2017). Identification of novel Ebola virus (EBOV) VP24 inhibitor from Indonesian natural products through in silico drug design approach. AIP Conf. Proc..

[68] Kwofie S.K., Broni E., Teye J., Quansah E., Issah I., Wilson M.D., Miller III W.A., Tiburu E.K., Bonney J.H. (2019). Pharmacoinformatics-based identification of potential bioactive compounds against Ebola virus protein VP24. Comput. Biol. Med..

[69] World Health Organization Categorization and Prioritization of Drugs for Consideration for Testing or Use in Patients Infected with Ebola. https://www.who.int/medicines/ebola-treatment/2015_0703TablesofEbolaDrugs.pdf?ua=1.

[70] Oestereich L., Lüdtke A., Wurr S., Rieger T., Muñoz-Fontela C., Günther S. (2014). Successful treatment of advanced Ebola virus infection with T-705 (favipiravir) in a small animal model. Antivir. Res..

[71] Warren T.K., Wells J., Panchal R.G., Stuthman K.S., Garza N.L., Van Tongeren S.A., Dong L., Retterer C.J., Eaton B.P., Pegoraro G. (2014). Protection against filovirus diseases by a novel broad-spectrum nucleoside analogue BCX4430. Nature.

[72] Warren T.K., Jordan R., Lo M.K., Ray A.S., Mackman R.L., Soloveva V., Siegel D., Perron M., Bannister R., Hui H.C. (2016). Therapeutic efficacy of the small molecule GS-5734 against Ebola virus in rhesus monkeys. Nature.

[73] Geisbert T.W., Hensley L.E., Kagan E., Yu E.Z., Geisbert J.B., Daddario-DiCaprio K., Fritz E.A., Jahrling P.B., McClintock K., Phelps J.R. (2006). Postexposure protection of guinea pigs against a lethal ebola virus challenge is conferred by RNA interference. J. Infect. Dis..

[74] Cong Y., Dyall J., DeWald L.E., Johnson J.C., Postnikova E., Zhou H., Gross R., Rojas O., Alexander I., Josleyn N. (2016). Evaluation of the activity of lamivudine and zidovudine against Ebola virus. PLoS ONE.

[75] van Hemert F.J., Zaaijer H.L., Berkhout B. (2015). In silico prediction of ebolavirus RNA polymerase inhibition by specific combinations of approved nucleotide analogues. J. Clin. Virol..

[76] Gehring G., Rohrmann K., Atenchong N., Mittler E., Becker S., Dahlmann F., Pöhlmann S., Vondran F.W., David S., Manns M.P. (2014). The clinically approved drugs amiodarone, dronedarone and verapamil inhibit filovirus cell entry. J. Antimicrob. Chemother..

[77] Salata C., Baritussio A., Munegato D., Calistri A., Ha H.R., Bigler L., Fabris F., Parolin C., Palù G., Mirazimi A. (2015). Amiodarone and metabolite MDEA inhibit Ebola virus infection by interfering with the viral entry process. Pathog. Dis..

[78] Madrid P.B., Panchal R.G., Warren T.K., Shurtleff A.C., Endsley A.N., Green C.E., Kolokoltsov A., Davey R., Manger I.D., Gilfillan L. (2015). Evaluation of Ebola Virus Inhibitors for Drug Repurposing. ACS Infect. Dis..

[79] Dyall J., Johnson J.C., Postnikova E., Cong Y., Zhou H., Gerhardt D.M., Michelotti J., Honko A.N., Kern S., DeWald L.E. (2018). In vitro and in vivo activity of amiodarone against Ebola virus. J. Infect. Dis..

[80] Kondratowicz A.S., Hunt C.L., Davey R.A., Cherry S., Maury W.J. (2013). AMP-activated protein kinase is required for the macropinocytic internalization of ebolavirus. J. Virol..

[81] Johnson J.C., Martinez O., Honko A.N., Hensley L.E., Olinger G.G., Basler C.F. (2014). Pyridinyl imidazole inhibitors of p38 MAP kinase impair viral entry and reduce cytokine induction by Zaire ebolavirus in human dendritic cells. Antivir. Res..

[82] Flint M., Chatterjee P., Lin D.L., McMullan L.K., Shrivastava-Ranjan P., Bergeron É., Lo M.K., Welch S.R., Nichol S.T., Tai A.W. (2019). A genome-wide CRISPR screen identifies N-acetylglucosamine-1-phosphate transferase as a potential antiviral target for Ebola virus. Nat. Commun..

[83] Yang S., Xu M., Lee E.M., Gorshkov K., Shiryaev S.A., He S., Sun W., Cheng Y.-S., Hu X., Tharappel A.M. (2018). Emetine inhibits Zika and Ebola virus infections through two molecular mechanisms: Inhibiting viral replication and decreasing viral entry. Cell Discov..

[84] Cheng H., Lear-Rooney C.M., Johansen L., Varhegyi E., Chen Z.W., Olinger G.G., Rong L. (2015). Inhibition of Ebola and Marburg virus entry by G protein-coupled receptor antagonists. J. Virol..

[85] Fan H., Du X., Zhang J., Zheng H., Lu X., Wu Q., Li H., Wang H., Shi Y., Gao G. (2017). Selective inhibition of Ebola entry with selective estrogen receptor modulators by disrupting the endolysosomal calcium. Sci. Rep..

[86] Mohr E.L., McMullan L.K., Lo M.K., Spengler J.R., Bergeron É., Albariño C.G., Shrivastava-Ranjan P., Chiang C.-F., Nichol S.T., Spiropoulou C.F. (2015). Inhibitors of cellular kinases with broad-spectrum antiviral activity for hemorrhagic fever viruses. Antivir. Res..

[87] Shurtleff A.C., Costantino J.A., Tritsch S.R., Retterer C., Spurgers K.B., Bavari S. (2014). HSPA5 is an essential host factor for Ebola virus infection. Antivir. Res..

[88] Smith D.R., McCarthy S., Chrovian A., Olinger G., Stossel A., Geisbert T.W., Hensley L.E., Connor J.H. (2010). Inhibition of heat-shock protein 90 reduces Ebola virus replication. Antivir. Res..

[89] Biedenkopf N., Lange-Grünweller K., Schulte F.W., Weißer A., Müller C., Becker D., Becker S., Hartmann R.K., Grünweller A. (2017). The natural compound silvestrol is a potent inhibitor of Ebola virus replication. Antivir. Res..

[90] Edwards M.R., Pietzsch C., Vausselin T., Shaw M.L., Bukreyev A., Basler C.F. (2015). High-Throughput Minigenome System for Identifying Small-Molecule Inhibitors of Ebola Virus Replication. ACS Infect. Dis.

[91] Huggins J., Zhang Z.X., Bray M. (1999). Antiviral drug therapy of filovirus infections: S-adenosylhomocysteine hydrolase inhibitors inhibit Ebola virus in vitro and in a lethal mouse model. J. Infect. Dis..

[92] Dyall J., Hart B.J., Postnikova E., Cong Y., Zhou H., Gerhardt D.M., Freeburger D., Michelotti J., Honko A.N., DeWald L.E. (2017). Interferon-beta and Interferon-gamma Are Weak Inhibitors of Ebola Virus in Cell-Based Assays. J. Infect. Dis..

[93] Jasenosky L.D., Cadena C., Mire C.E., Borisevich V., Haridas V., Ranjbar S., Nambu A., Bavari S., Soloveva V., Sadukhan S. (2019). The FDA-Approved Oral Drug Nitazoxanide Amplifies Host Antiviral Responses and Inhibits Ebola Virus. iScience.

[94] Kinch M.S., Yunus A.S., Lear C., Mao H., Chen H., Fesseha Z., Luo G., Nelson E.A., Li L., Huang Z. (2009). FGI-104: A broad-spectrum small molecule inhibitor of viral infection. Am. J. Transl. Res..

[95] Geisbert T.W., Hensley L.E., Jahrling P.B., Larsen T., Geisbert J.B., Paragas J., Young H.A., Fredeking T.M., Rote W.E., Vlasuk G.P. (2003). Treatment of Ebola virus infection with a recombinant inhibitor of factor VIIa/tissue factor: A study in rhesus monkeys. Lancet.

[96] Hensley L.E., Stevens E.L., Yan S.B., Geisbert J.B., Macias W.L., Larsen T., Daddario-DiCaprio K.M., Cassell G.H., Jahrling P.B., Geisbert T.W. (2007). Recombinant human activated protein C for the postexposure treatment of Ebola hemorrhagic fever. J. Infect. Dis..

[97] Warfield K.L., Swenson D.L., Olinger G.G., Nichols D.K., Pratt W.D., Blouch R., Stein D.A., Aman M.J., Iversen P.L., Bavari S. (2006). Gene-specific countermeasures against Ebola virus based on antisense phosphorodiamidate morpholino oligomers. PLoS Pathog..

[98] Warren T.K., Warfield K.L., Wells J., Swenson D.L., Donner K.S., Van Tongeren S.A., Garza N.L., Dong L., Mourich D.V., Crumley S. (2010). Advanced antisense therapies for postexposure protection against lethal filovirus infections. Nat. Med..

[99] Richardson J.S., Wong G., Pillet S., Schindle S., Ennis J., Turner J., Strong J.E., Kobinger G.P. (2011). Evaluation of Different Strategies for Post-Exposure Treatment of Ebola Virus Infection in Rodents. J. Bioterror. Biodef..

[100] Qiu X., Wong G., Fernando L., Audet J., Bello A., Strong J., Alimonti J.B., Kobinger G.P. (2013). mAbs and Ad-vectored IFN-alpha therapy rescue Ebola-infected nonhuman primates when administered after the detection of viremia and symptoms. Sci. Transl. Med..

[101] Yu D.S., Weng T.H., Shen L., Wu X.X., Hu C.Y., Wang F.X.C., Wu Z.G., Wu H.B., Wu N.P., Li L.J. (2018). Development and Characterization of Neutralizing Antibodies Against Zaire Ebolavirus Glycoprotein and Protein 40. Cell. Physiol. Biochem..

[102] McCarthy S.D., Majchrzak-Kita B., Racine T., Kozlowski H.N., Baker D.P., Hoenen T., Kobinger G.P., Fish E.N., Branch D.R. (2016). A Rapid Screening Assay Identifies Monotherapy with Interferon-ss and Combination Therapies with Nucleoside Analogs as Effective Inhibitors of Ebola Virus. PLoS Negl. Trop. Dis..

[103] Sun W., He S., Martínez-Romero C., Kouznetsova J., Tawa G., Xu M., Shinn P., Fisher E.G., Long Y., Motabar O. (2017). Synergistic drug combination effectively blocks Ebola virus infection. Antivir. Res..

[104] Dyall J., Nelson E.A., DeWald L.E., Guha R., Zhou H., Postnikova E., Logue J., Vargas W.M., Gross R., Michelotti J. (2018). Identification of combinations of approved drugs with synergistic activity against Ebola virus in cell cultures. J. Infect. Dis..

[105] Du X., Zuo X., Meng F., Wu F., Zhao X., Li C., Cheng G., Qin F.X.-F. (2020). Combinatorial screening of a panel of FDA-approved drugs identifies several candidates with anti-Ebola activities. Biochem. Biophys. Res. Commun..

[106] Anantpadma M., Kouznetsova J., Wang H., Huang R., Kolokoltsov A., Guha R., Lindstrom A.R., Shtanko O., Simeonov A., Maloney D.J. (2016). Large-Scale Screening and Identification of Novel Ebola Virus and Marburg Virus Entry Inhibitors. Antimicrob. Agents Chemother..

[107] Yermolina M.V., Wang J., Caffrey M., Rong L.L., Wardrop D.J. (2011). Discovery, synthesis, and biological evaluation of a novel group of selective inhibitors of filoviral entry. J. Med. Chem..

[108] Wang Y., Cui R., Li G., Gao Q., Yuan S., Altmeyer R., Zou G. (2016). Teicoplanin inhibits Ebola pseudovirus infection in cell culture. Antivir. Res..

[109] Long J., Wright E., Molesti E., Temperton N., Barclay W. (2015). Antiviral therapies against Ebola and other emerging viral diseases using existing medicines that block virus entry. F1000Research.

[110] Kouznetsova J., Sun W., Martinez-Romero C., Tawa G., Shinn P., Chen C.Z., Schimmer A., Sanderson P., McKew J.C., Zheng W. (2014). Identification of 53 compounds that block Ebola virus-like particle entry via a repurposing screen of approved drugs. Emerg. Microbes Infect..

[111] Falzarano D., Safronetz D., Prescott J., Marzi A., Feldmann F., Feldmann H. (2015). Lack of protection against ebola virus from chloroquine in mice and hamsters. Emerg. Infect. Dis.

[112] Qiu X., Kroeker A., He S., Kozak R., Audet J., Mbikay M., Chrétien M. (2016). Prophylactic efficacy of quercetin 3-β-Od-glucoside against Ebola virus infection. Antimicrob. Agents Chemother..

[113] Marzi A., Yoshida R., Miyamoto H., Ishijima M., Suzuki Y., Higuchi M., Matsuyama Y., Igarashi M., Nakayama E., Kuroda M. (2012). Protective efficacy of neutralizing monoclonal antibodies in a nonhuman primate model of Ebola hemorrhagic fever. PLoS ONE.

[114] Qiu X., Audet J., Wong G., Pillet S., Bello A., Cabral T., Strong J.E., Plummer F., Corbett C.R., Alimonti J.B. (2012). Successful treatment of ebola virus-infected cynomolgus macaques with monoclonal antibodies. Sci. Transl. Med..

[115] Limberis M.P., Tretiakova A., Nambiar K., Wong G., Racine T., Crosariol M., Xiangguo Q., Kobinger G., Wilson J.M. (2016). Adeno-Associated Virus Serotype 9-Expressed ZMapp in Mice Confers Protection Against Systemic and Airway-Acquired Ebola Virus Infection. J. Infect. Dis..

[116] Robert M.A., Nassoury N., Chahal P.S., Venne M.H., Racine T., Qiu X., Kobinger G., Kamen A., Gilbert R., Gaillet B. (2018). Gene Transfer of ZMapp Antibodies Mediated by Recombinant Adeno-Associated Virus Protects Against Ebola Infections. Hum. Gene.

[117] van Lieshout L.P., Soule G., Sorensen D., Frost K.L., He S., Tierney K., Safronetz D., Booth S.A., Kobinger G.P., Qiu X. (2018). Intramuscular Adeno-Associated Virus-Mediated Expression of Monoclonal Antibodies Provides 100% Protection Against Ebola Virus Infection in Mice. J. Infect. Dis..

[118] Oswald W.B., Geisbert T.W., Davis K.J., Geisbert J.B., Sullivan N.J., Jahrling P.B., Parren P.W., Burton D.R. (2007). Neutralizing antibody fails to impact the course of Ebola virus infection in monkeys. PLoS Pathog..

[119] Brannan J.M., He S., Howell K.A., Prugar L.I., Zhu W., Vu H., Shulenin S., Kailasan S., Raina H., Wong G. (2019). Post-exposure immunotherapy for two ebolaviruses and Marburg virus in nonhuman primates. Nat. Commun..

[120] Wec A.Z., Bornholdt Z.A., He S., Herbert A.S., Goodwin E., Wirchnianski A.S., Gunn B.M., Zhang Z., Zhu W., Liu G. (2019). Development of a Human Antibody Cocktail that Deploys Multiple Functions to Confer Pan-Ebolavirus Protection. Cell Host Microbe.

[121] Bornholdt Z.A., Herbert A.S., Mire C.E., He S., Cross R.W., Wec A.Z., Abelson D.M., Geisbert J.B., James R.M., Rahim M.N. (2019). A Two-Antibody Pan-Ebolavirus Cocktail Confers Broad Therapeutic Protection in Ferrets and Nonhuman Primates. Cell Host Microbe.

[122] Smither S.J., Eastaugh L.S., Steward J.A., Nelson M., Lenk R.P., Lever M.S. (2014). Post-exposure efficacy of Oral T-705 (Favipiravir) against inhalational Ebola virus infection in a mouse model. Antivir. Res..

[123] Guedj J., Piorkowski G., Jacquot F., Madelain V., Nguyen T.H.T., Rodallec A., Gunther S., Carbonnelle C., Mentré F., Raoul H. (2018). Antiviral efficacy of favipiravir against Ebola virus: A translational study in cynomolgus macaques. PLoS Med..

[124] Bixler S.L., Bocan T.M., Wells J., Wetzel K.S., Van Tongeren S.A., Dong L., Garza N.L., Donnelly G., Cazares L.H., Nuss J. (2018). Efficacy of favipiravir (T-705) in nonhuman primates infected with Ebola virus or Marburg virus. Antivir. Res..

[125] Warren T., MacLennan S., Mathis A., Giuliano E., Taylor R., Sheridan W. (2017). Efficacy of Galidesivir against Ebola Virus Disease in Rhesus Monkeys. Open Forum Infect. Dis..

[126] Bray M., Driscoll J., Huggins J.W. (2000). Treatment of lethal Ebola virus infection in mice with a single dose of an S-adenosyl-l-homocysteine hydrolase inhibitor. Antivir. Res..

[127] Bray M., Raymond J.L., Geisbert T., Baker R.O. (2002). 3-Deazaneplanocin A induces massively increased interferon-α production in Ebola virus-infected mice. Antivir. Res..

[128] Rhein B.A., Powers L.S., Rogers K., Anantpadma M., Singh B.K., Sakurai Y., Bair T., Miller-Hunt C., Sinn P., Davey R.A. (2015). Interferon-gamma Inhibits Ebola Virus Infection. PLoS Pathog..

[129] Jahrling P.B., Geisbert T.W., Geisbert J.B., Swearengen J.R., Bray M., Jaax N.K., Huggins J.W., LeDuc J.W., Peters C.J. (1999). Evaluation of immune globulin and recombinant interferon-alpha2b for treatment of experimental Ebola virus infections. J. Infect. Dis..

[130] Smith L.M., Hensley L.E., Geisbert T.W., Johnson J., Stossel A., Honko A., Yen J.Y., Geisbert J., Paragas J., Fritz E. (2013). Interferon-beta therapy prolongs survival in rhesus macaque models of Ebola and Marburg hemorrhagic fever. J. Infect. Dis..

[131] Geisbert T.W., Lee A.C., Robbins M., Geisbert J.B., Honko A.N., Sood V., Johnson J.C., de Jong S., Tavakoli I., Judge A. (2010). Postexposure protection of non-human primates against a lethal Ebola virus challenge with RNA interference: A proof-of-concept study. Lancet.

[132] Dowall S.D., Bosworth A., Watson R., Bewley K., Taylor I., Rayner E., Hunter L., Pearson G., Easterbrook L., Pitman J. (2015). Chloroquine inhibited Ebola virus replication in vitro but failed to protect against infection and disease in the in vivo guinea pig model. J. Gen. Virol..

[133] Prins K.C., Binning J.M., Shabman R.S., Leung D.W., Amarasinghe G.K., Basler C.F. (2010). Basic residues within the ebolavirus VP35 protein are required for its viral polymerase cofactor function. J. Virol..

[134] Basler C.F., Wang X., Mühlberger E., Volchkov V., Paragas J., Klenk H.-D., García-Sastre A., Palese P. (2000). The Ebola virus VP35 protein functions as a type I IFN antagonist. Proc. Natl. Acad. Sci. USA.

[135] Jasenosky L.D., Neumann G., Lukashevich I., Kawaoka Y. (2001). Ebola Virus VP40-Induced Particle Formation and Association with the Lipid Bilayer. J. Virol..

[136] Hoenen T., Jung S., Herwig A., Groseth A., Becker S. (2010). Both matrix proteins of Ebola virus contribute to the regulation of viral genome replication and transcription. Virology.

[137] Bornholdt Z.A., Noda T., Abelson D.M., Halfmann P., Wood M.R., Kawaoka Y., Saphire E.O. (2013). Structural Rearrangement of Ebola Virus VP40 Begets Multiple Functions in the Virus Life Cycle. Cell.

[138] Hoenen T., Volchkov V., Kolesnikova L., Mittler E., Timmins J., Ottmann M., Reynard O., Becker S., Weissenhorn W. (2005). VP40 Octamers Are Essential for Ebola Virus Replication. J. Virol..

[139] Maruyama T., Rodriguez L.L., Jahrling P.B., Sanchez A., Khan A.S., Nichol S.T., Peters C.J., Parren P.W., Burton D.R. (1999). Ebola virus can be effectively neutralized by antibody produced in natural human infection. J. Virol..

[140] Bornholdt Z.A., Turner H.L., Murin C.D., Li W., Sok D., Souders C.A., Piper A.E., Goff A., Shamblin J.D., Wollen S.E. (2016). Isolation of potent neutralizing antibodies from a survivor of the 2014 Ebola virus outbreak. Science.

[141] Feldmann H., Jones S.M., Daddario-DiCaprio K.M., Geisbert J.B., Stroher U., Grolla A., Bray M., Fritz E.A., Fernando L., Feldmann F. (2007). Effective post-exposure treatment of Ebola infection. PLoS Pathog..

[142] Henao-Restrepo A.M., Longini I.M., Egger M., Dean N.E., Edmunds W.J., Camacho A., Carroll M.W., Doumbia M., Draguez B., Duraffour S. (2015). Efficacy and effectiveness of an rVSV-vectored vaccine expressing Ebola surface glycoprotein: Interim results from the Guinea ring vaccination cluster-randomised trial. Lancet.

[143] Qiu X., Audet J., Wong G., Fernando L., Bello A., Pillet S., Alimonti J.B., Kobinger G.P. (2013). Sustained protection against Ebola virus infection following treatment of infected nonhuman primates with ZMAb. Sci. Rep..

[144] Lyon G.M., Mehta A.K., Varkey J.B., Brantly K., Plyler L., McElroy A.K., Kraft C.S., Towner J.S., Spiropoulou C., Ströher U. (2014). Clinical care of two patients with Ebola virus disease in the United States. N. Engl. J. Med..

[145] The PREVAIL II Writing Group (2016). A Randomized, Controlled Trial of ZMapp for Ebola Virus Infection. N. Engl. J. Med..

[146] Mulangu S., Dodd L.E., Davey Jr R.T., Tshiani Mbaya O., Proschan M., Mukadi D., Lusakibanza Manzo M., Nzolo D., Tshomba Oloma A., Ibanda A. (2019). A randomized, controlled trial of Ebola virus disease therapeutics. N. Engl. J. Med..

[147] Ji X., Olinger G.G., Aris S., Chen Y., Gewurz H., Spear G.T. (2005). Mannose-binding lectin binds to Ebola and Marburg envelope glycoproteins, resulting in blocking of virus interaction with DC-SIGN and complement-mediated virus neutralization. J. Gen. Virol..

[148] Zhao Y., Ren J., Harlos K., Jones D.M., Zeltina A., Bowden T.A., Padilla-Parra S., Fry E.E., Stuart D.I. (2016). Toremifene interacts with and destabilizes the Ebola virus glycoprotein. Nature.

[149] Zhao Y., Ren J., Fry E.E., Xiao J., Townsend A.R., Stuart D.I. (2018). Structures of Ebola Virus Glycoprotein Complexes with Tricyclic Antidepressant and Antipsychotic Drugs. J. Med. Chem..

[150] Ren J., Zhao Y., Fry E.E., Stuart D.I. (2018). Target Identification and Mode of Action of Four Chemically Divergent Drugs against Ebolavirus Infection. J. Med. Chem..

[151] Biedenkopf N., Lier C., Becker S., Lyles D.S. (2016). Dynamic Phosphorylation of VP30 Is Essential for Ebola Virus Life Cycle. J. Virol..

[152] Ilinykh P.A., Tigabu B., Ivanov A., Ammosova T., Obukhov Y., Garron T., Kumari N., Kovalskyy D., Platonov M.O., Naumchik V.S. (2014). Role of protein phosphatase 1 in dephosphorylation of Ebola virus VP30 protein and its targeting for the inhibition of viral transcription. J. Biol. Chem..

[153] Dunning J., Kennedy S.B., Antierens A., Whitehead J., Ciglenecki I., Carson G., Kanapathipillai R., Castle L., Howell-Jones R., Pardinaz-Solis R. (2016). Experimental treatment of Ebola virus disease with brincidofovir. PLoS ONE.

[154] Kerber R., Lorenz E., Duraffour S., Sissoko D., Rudolf M., Jaeger A., Cisse S.D., Camara A.-M., Miranda O., Castro C.M. (2019). Laboratory Findings, Compassionate Use of Favipiravir, and Outcome in Patients With Ebola Virus Disease, Guinea, 2015—A Retrospective Observational Study. J. Infect. Dis..

[155] BioCryst BioCryst Completes Phase 1 Clinical Trial of Galidesivir. https://ir.biocryst.com/news-releases/news-release-details/biocryst-completes-phase-1-clinical-trial-galidesivir.

[156] Turone F. (2014). Doctors trial amiodarone for Ebola in Sierra Leone. BMJ.

[157] Decroly E., Ferron F., Lescar J., Canard B. (2011). Conventional and unconventional mechanisms for capping viral mRNA. Nat. Rev. Microbiol..

[158] Konde M.K., Baker D.P., Traore F.A., Sow M.S., Camara A., Barry A.A., Mara D., Barry A., Cone M., Kaba I. (2017). Interferon beta-1a for the treatment of Ebola virus disease: A historically controlled, single-arm proof-of-concept trial. PLoS ONE.

[159] Hoenen T., Safronetz D., Groseth A., Wollenberg K., Koita O., Diarra B., Fall I., Haidara F., Diallo F., Sanogo M. (2015). Mutation rate and genotype variation of Ebola virus from Mali case sequences. Science.

[160] Kugelman J.R., Kugelman-Tonos J., Ladner J.T., Pettit J., Keeton C.M., Nagle E.R., Garcia K.Y., Froude J.W., Kuehne A.I., Kuhn J.H. (2015). Emergence of Ebola virus escape variants in infected nonhuman primates treated with the MB-003 antibody cocktail. Cell Rep..

[161] PubChem Digitoxin. https://pubchem.ncbi.nlm.nih.gov/compound/Digitoxin.

[162] PubChem Sertraline. https://pubchem.ncbi.nlm.nih.gov/compound/68617.

